# A familial risk enriched cohort as a platform for testing early interventions to prevent severe mental illness

**DOI:** 10.1186/s12888-014-0344-2

**Published:** 2014-12-02

**Authors:** Rudolf Uher, Jill Cumby, Lynn E MacKenzie, Jessica Morash-Conway, Jacqueline M Glover, Alice Aylott, Lukas Propper, Sabina Abidi, Alexa Bagnell, Barbara Pavlova, Tomas Hajek, David Lovas, Kathleen Pajer, William Gardner, Adrian Levy, Martin Alda

**Affiliations:** Capital District Health Authority, Halifax, Nova Scotia Canada; IWK Health Centre, Halifax, Nova Scotia Canada; Department of Psychiatry, Dalhousie University, Halifax, Nova Scotia Canada; Department of Psychology and Neuroscience, Dalhousie University, Halifax, Nova Scotia Canada; Department of Public Health and Epidemiology, Dalhousie University, Halifax, Nova Scotia Canada

**Keywords:** Severe mental illness, Schizophrenia, Bipolar disorder, Major depressive disorder, Cohort study, High-risk offspring, Targeted prevention, Early interventions

## Abstract

**Background:**

Severe mental illness (SMI), including schizophrenia, bipolar disorder and severe depression, is responsible for a substantial proportion of disability in the population. This article describes the aims and design of a research study that takes a novel approach to targeted prevention of SMI. It is based on the rationale that early developmental antecedents to SMI are likely to be more malleable than fully developed mood or psychotic disorders and that low-risk interventions targeting antecedents may reduce the risk of SMI.

**Methods/Design:**

Families Overcoming Risks and Building Opportunities for Well-being (FORBOW) is an accelerated cohort study that includes a large proportion of offspring of parents with SMI and embeds intervention trials in a cohort multiple randomized controlled trial (cmRCT) design. Antecedents are conditions of the individual that are distressing but not severely impairing, predict SMI with moderate-to-large effect sizes and precede the onset of SMI by at least several years. FORBOW focuses on the following antecedents: affective lability, anxiety, psychotic-like experiences, basic symptoms, sleep problems, somatic symptoms, cannabis use and cognitive delay. Enrolment of offspring over a broad age range (0 to 21 years) will allow researchers to draw conclusions on a longer developmental period from a study of shorter duration. Annual assessments cover a full range of psychopathology, cognitive abilities, eligibility criteria for interventions and outcomes. Pre-emptive early interventions (PEI) will include skill training for parents of younger children and courses in emotional well-being skills based on cognitive behavioural therapy for older children and youth. A sample enriched for familial risk of SMI will enhance statistical power for testing the efficacy of PEI.

**Discussion:**

FORBOW offers a platform for efficient and unbiased testing of interventions selected according to best available evidence. Since few differences exist between familial and ’sporadic’ SMI, the same interventions are likely to be effective in the general population. Comparison of short-term efficacy of PEI on antecedents and the long term efficacy for preventing the onset of SMI will provide an experimental test of the etiological role of antecedents in the development of SMI.

**Electronic supplementary material:**

The online version of this article (doi:10.1186/s12888-014-0344-2) contains supplementary material, which is available to authorized users.

## Background

Severe mental illness (SMI), including schizophrenia, bipolar disorder and severe depression, is responsible for a substantial proportion of disability in the population. Current treatments may ameliorate the course of SMI, but do not provide a cure. Therefore, prevention of SMI is a public health priority. To date, early interventions have focussed on the prodromal stage shortly preceding the onset of SMI [1]. These interventions have had some notable successes, including halving the short-term risk of developing SMI with a purely psychological approach [2]. However, relatively poor long-term functional outcomes [3] suggest that interventions in the prodromal stage may come too late to normalize the developmental trajectory. Therefore, pre-emptive early interventions (PEI) at earlier stages of development may need to be considered [4]. Because the familial and environmental risk factors for mood and psychotic disorders largely overlap [5],[6] and because early antecedents are less specific than prodrome [7], PEI may need to focus on broader categories, such as SMI, rather than a specific diagnosis.

PEI can be informed by what is known about SMI. First, SMI runs in families and the risk varies with the degree of biological relatedness to an affected individual. The familial risk is partly diagnostically specific: a son or daughter of a parent with schizophrenia will have approximately eight-fold increased risk of developing schizophrenia, but also a two-fold increased risk of developing bipolar disorder or depression [8]. Overall, one in three offspring of parents with SMI will develop a major mood or psychotic disorder by early adulthood [8]. Molecular genetic variants also largely overlap between mood and psychotic disorders [6],[9],[10]. Second, SMI may be more predictable than previously thought. Longitudinal studies of representative population cohorts suggest that most cases of SMI are preceded by earlier antecedents. Antecedents including delays in cognitive development, affective lability, anxiety, sleep problems, psychotic-like experiences and basic symptoms are detectable in childhood or adolescence, and predict the onset of SMI 4 to 15 years later with substantial effect sizes [11]-[15]. This means that many cases of SMI can be predicted before the prodromal stage to enable targeted PEI. Third, the genetic and neurodevelopmental risk factors for SMI are malleable [16],[17]. A Finnish adoption study found that high quality parenting reduced the risk of psychosis in adopted offspring of biological mothers with schizophrenia to a level comparable to adoptees from mothers with no mental illness [18]. Longitudinal neuroimaging studies show brain abnormalities in individuals at familial risk at age 6-to-14 years that normalize by age 17 in those who do not develop early onset SMI but persist in those affected with SMI [19],[20]. Taken together, these three areas of knowledge indicate that the risk of SMI is measurable and modifiable in childhood and adolescence.

We propose that antecedents in combination with family history of SMI present an opportunity for developing and testing PEI at earlier stages of development than current ‘early interventions’. The use of a sample enriched for family history of SMI will increase statistical power for testing interventions’ effects on SMI risk because of the high base risk of developing SMI [8]. The incomplete penetrance of multiple weak genetic risk variants means that familial and sporadic cases of SMI are unlikely to be fundamentally different [21]. Therefore, interventions developed in familial high-risk context are likely to generalize to broader target populations. Antecedents detected at earlier stages of development are likely to be less specific and less impairing than prodrome or full-blown SMI [1],[22]. Therefore, PEI may have to target the risk of mental illness in general or broader groupings like SMI rather than narrow diagnostic categories [5],[6]. Following the principles of staging and proportionality of interventions to current degree of problems [1],[22], the most acceptable and practical PEI will be interventions that carry low burden to participants, have very low risk of adverse effects, and are likely to be beneficial irrespective of whether a given individual was going to develop SMI or not. Even at the prodromal stage, low-risk psychological interventions were at least as effective as antipsychotic medication that carries a high burden of adverse effects [2],[23],[24]. Another low-risk intervention that has shown efficacy in the prodromal stage is dietary supplementation with polyunsaturated fatty acids [25]. Therefore, psychological interventions and dietary supplements are likely to be among the most practical and acceptable PEI.

In this article we outline the design and methods of a familial risk enriched cohort study that aims to test the efficacy of PEI for preventing SMI.

## Methods/Design

### Aims

This study has two related aims:

1. Explore the role of selected psychopathological and cognitive antecedents in the development of severe mental illness.
2. Evaluate the efficacy of antecedent-focussed pre-emptive early interventions in reducing psychopathology, improving functioning and preventing SMI.

### Design

**Figure 1:**
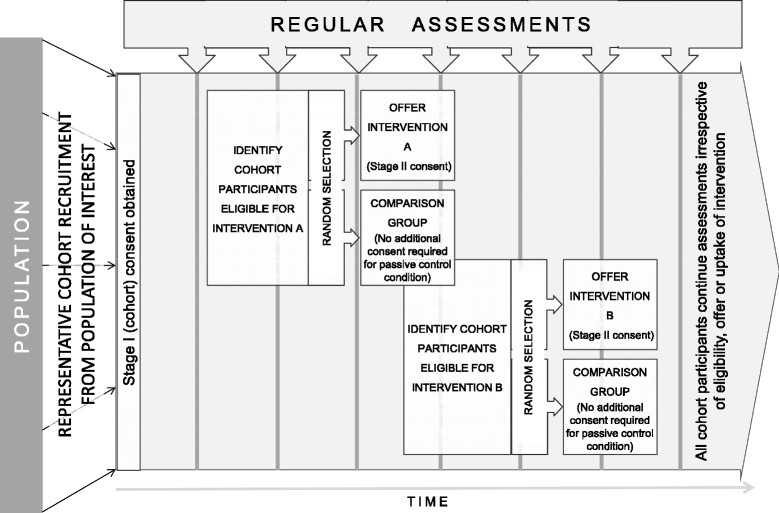
**Cohort-multiple randomized controlled trial design.**

Families Overcoming Risks and Building Opportunities for Well-being (FORBOW) is an accelerated cohort study enriched in familial risk for SMI. The cohort is designed as a platform that can incorporate randomized controlled trials of PEI in a cohort multiple randomized controlled trial (cmRCT) design (Figure 1) [26]. In a cmRCT, participants entering the cohort provide consent for their information to be used in the evaluation of interventions. Eligibility criteria and outcomes are assessed as part of regular cohort follow-ups. Some eligible participants are randomly selected to be offered an intervention. Those who are randomly selected for intervention are approached with an offer of intervention and provide a separate informed consent for the intervention only. Those who are not randomly selected to be offered the intervention do not need to sign a second consent since they are providing all measures as part of their participation in the cohort study. This design is more pragmatic than traditional randomized controlled trials since it mirrors the practice of offering a preventive intervention to non-treatment seeking participants and saves the participants from the unnatural and possibly harmful effect of being allocated to a control group after hearing about the details of a potentially beneficial intervention. Over time, cohort participants can be randomly selected for one or more interventions. Selection for each intervention is independent, allowing researchers to examine effects of each intervention independently as well as effects of sequential interventions. The accelerated character means that participants can enter the study at a range of ages and the cohort will allow drawing conclusions about a longer developmental period over a shorter study duration, taking advantage of intra-individual continuities and inter-individual differences [27]-[29]. Accelerated cohort is also suitable for interventions, since the participants are gradually moving through the age window of eligibility, optimizing the use of therapeutic resources and allowing to complete relatively large intervention studies with small intervention teams.

In FORBOW, the regular assessments occur in 12 month intervals. At each follow-up, we collect measures to assess the eligibility for intervention as well as primary and secondary outcomes. Separate teams assess offspring and parents. The researchers who assess the offspring are blind to the diagnosis of parents and vice versa. In 2013, the FORBOW study was launched in a single centre in Halifax, Nova Scotia. With inclusion of additional centres, FORBOW is likely to become a multi-centre study.

### Focus on severe mental illness

Several lines of research suggest that study of mental illness should not be limited to one diagnostic category and that there is an advantage in studying broader categories in less selected samples. Conditions that are classified as separate diagnoses share most genetic and environmental risk factors [5],[6],[8]. In addition, the early antecedents to mental illness may be less diagnosis-specific and the most pragmatic aim is prevention of any SMI rather than one specific disorder [1],[22]. Therefore, the primary focus of the FORBOW study is the broad category of SMI. Our definition of SMI comprises major psychotic and mood disorders that typically start in late adolescence or early adulthood and reach a severity that requires inpatient or intensive psychiatric care. We include in the definition of SMI all cases of schizophrenia, schizoaffective disorder and bipolar disorder type I. We include cases of major depressive disorder and bipolar disorder type II if they fulfill two or more severity criteria: (1) severity that requires hospital admission, (2) recurrence (3 or more episodes within 10 years), (3) chronicity (symptoms present for most days over two years or longer with no remissions lasting 2 months or longer), (4) psychotic symptoms or (5) a life-threatening suicide attempt. These severity criteria are designed so that cases of broadly defined disorders are included if they reach the degree of severity implicit in the concept of SMI.

### Participants, inclusion and exclusion criteria

FORBOW enrolls offspring of parents with SMI (FHR, family high-risk offspring), and offspring of healthy parents matched on neighbourhood and demographic factors (CO, comparison offspring). FHR are recruited by referrals from adult mental health services, by clinicians who treat parents with SMI. The recruitment materials emphasise that all biological children should be invited to participate, irrespective of whether or not they live with the biological parent and whether or not there are any concerns about their mental health. In several mental health services across Nova Scotia, a systematic recruitment procedure is in place where all patients are asked about the number and age of biological children and patients with SMI and one or more biological children in the eligible age range are referred to FORBOW. Partnership with the Department of Community Services, Nova Scotia, allows following up children who are not in the care of their biological parents. CO are recruited through two pathways: (1) acquaintance referrals, targeting families living in the same neighbourhood and having children of the same age as the FHR; (2) school recruitment by approaching parents of children in the same geographic areas where FHR are enrolled. Both ways of recruitment are designed to obtain a sample of CO who are similar to FHR offspring in terms of neighbourhood, school and socioeconomic status. At the time of writing, FORBOW is enrolling offspring between ages 3 years and 21 years. A planned downward extension (FORBOW-ELF, Early Life Focus) will include offspring below age 3. All offspring continue to be followed up until age 27, to cover the highest risk period for SMI onset. To maximize generalizability, FORBOW assumes broad inclusion and minimal exclusion criteria. All biological offspring in the eligible age range can participate in the study provided that at least one of their biological parents is available for assessment and that the offspring or their legal guardian provides a valid informed consent. Multiple offspring from the same family can enrol. Exclusion criteria are acquired brain injury or intellectual disability of a degree that makes all or most assessments invalid. Offspring with milder intellectual disability, autism or attention-deficit hyperactivity disorder can participate, but the range of assessments may be reduced, given their attention, comprehension and communication abilities. Biological parents and other caregivers are also FORBOW participants.

### Sample size and power calculation

**Figure 2:**
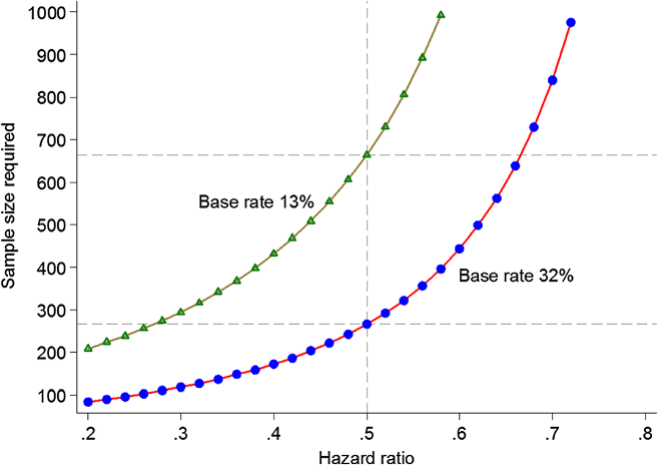
**Sample size requirements to detect the effect of preventive intervention.** On the y axis is the sample size required to detect an effect of intervention as statistically significant with a power of 80%. On the x axis is the hazard ratio reflecting how much an intervention reduces the risk of severe mental illness. The two plotted lines demonstrate the dependence of sample size requirements on the risk of developing severe mental illness in the absence of an intervention (base rate). In a high-risk population of offspring of parents with severe mental illness (base rate 32%), the sample size required is 2.5 times lower than in the general population (base rate 13%).

It is our aim to halve the risk of SMI by providing PEIs. The sample size required to detect such effects with adequate statistical power depends on the base risk of developing SMI in the absence of intervention (Figure 2). Based on a meta-analysis of published familial high-risk studies, the risk that an offspring of a parent with SMI develops a major mood or psychotic disorder by early adulthood is 32%, compared to 13% in the general population [8]. Consequently, testing interventions in high-risk offspring reduces the sample size requirements 2.5-fold compared to general population samples. Assuming a 3:1 ratio of FHR and CO, and a 15% attrition on follow-up, a sample of 316 (158 receiving intervention and 158 in a control arm) is required to detect an effect of intervention that halves the risk of SMI as statistically significant (p < 0.05) in a survival analysis with a power of 80%. Pilot data suggest that approximately 50% participants may be eligible for interventions. Therefore, FORBOW aims to recruit 632 participants.

### Antecedents

#### Definition of antecedents

We define antecedents as conditions of the individual, that predict SMI and precede its onset by at least several years. The requirement that antecedents are conditions of the individual excludes external risk factors, such as poverty, violence, childhood maltreatment, urban upbringing and exposure to toxins or infections. We select antecedents that have robust evidence for predicting SMI with a moderate to large effect size (risk ratio greater than 2), so that intervention efforts are not directed towards risk factors that have only a trivial effect on risk. Antecedents are typically distressing for the individual or the family, motivating an intervention irrespective of whether they augur more severe problems or not. Antecedents are typically not severely impairing and occur before the individual has missed out on major developmental opportunities. The typical lag between an antecedent and the onset of SMI can be inferred from published cohort studies. The requirement that an antecedent typically precedes the onset of SMI by at least several years (operationalized as ≥4 years) is intended to focus intervention efforts to earlier stages where the developmental trajectories can be influenced with a smaller investment to a greater effect [30],[31]. Following this definition, we reviewed the literature and selected antecedents that are listed in Table 1 and described below.

**Table 1:** **Antecedents to severe mental illness**

Antecedent	Intervention	Age (years)
Affective lability	Parenting	4 to 9
	Cognitive-behavioural skills	9 to 21
Anxiety	Parenting	6 to 9
	Cognitive-behavioural skills	9 to 21
Psychotic-like experiences	Cognitive-behavioural skills	7 to 21
	Dietary (polyunsaturated fatty acids)	7 to 21
Basic symptoms	Cognitive-behavioural skills	9 to 21
	Dietary (polyunsaturated fatty acids)	7 to 21
Cognitive delay	Parenting, cognitive training	4 to 9
	Dietary (polyunsaturated fatty acids)	4 to 21
Somatic symptoms	Parenting	4 to 9
	Cognitive-behavioural skills, mindfulness	9 to 21
Sleep problems	Parenting	4 to 9
	Cognitive-behavioural skills, mindfulness	9 to 21
Cannabis use	Personality targeted cognitive-behavioural intervention	11 to 21

Next to each antecedent, we list potential interventions and age range for antecedent assessment and intervention.

#### Affective lability

Affective lability (AL) is the propensity to experience strong and sudden changes in mood that are seen by others as unpredictable [32],[33]. AL, measured by self-report, parent-report, momentary experience sampling or in response to experimental provocation is increased in individuals with bipolar disorder and in offspring of parents with bipolar disorder [34]-[38]. Increased AL persists in full remission and separates individuals with bipolar disorder from those with other diagnoses [36],[37]. AL predicts development of bipolar disorder in prospective studies [39]-[41]. Therefore, AL may be an antecedent to bipolar disorder [36],[37],[42],[43]. Increased AL has also been reported in major depressive disorder [42]. The minimal available data suggest that AL may also be a feature of schizophrenia [44].

#### Anxiety

Anxiousness and anxiety disorders are common antecedents to many types of mental illness [11],[12],[45]-[47]. The rate of anxiety disorders is doubled among offspring of parents with bipolar disorder or depression [8],[11],[41],[48]-[52]. In the context of family history, anxiety in childhood or adolescence confers very high risk of bipolar disorder and depression [11],[48]-[50]. Anxiety disorders precede the development of the first major episode of SMI by on average eight years [11]. Anxiety disorders respond well to cognitive behavioural therapy (CBT) [53],[54]. The combined evidence suggests that anxiety disorders may represent a modifiable stage in the development of mood disorders [11],[41]. The relationship between anxiety and schizophrenia is less well understood. Some evidence supports a continuum from anxiety to psychosis [55],[56]. While population-based registry studies suggest a familial association between anxiety disorders and schizophrenia [57], a meta-analysis of family high risk studies found sparse data and no evidence of association [8].

#### Psychotic-like experiences

While schizophrenia and other psychotic disorders typically onset in late adolescence or early adulthood, isolated psychotic symptoms are frequently experienced in childhood. These early symptoms typically include hallucinations and, in the absence of psychotic disorder, are commonly referred to as ‘psychotic-like experiences’ (PLE). PLE are reported by 5% adults, 7.5% adolescents and up to 17% children in the general population [58]-[60]. Psychotic symptoms in childhood and adolescence predict SMI in adulthood with moderately high specificity [15],[60]-[62]. Temporal course of PLE may be important, with persistent PLE being more predictive of SMI than transitory PLE [63]-[65]. Since childhood PLE have overlapping aetiological factors with full-blown psychosis [66], they can be conceptualized as antecedents and represent a potential target for PEI. There is evidence that PLE are more frequent in offspring of parents with SMI [66], but PLE have not been systematically evaluated in familial high-risk setting.

#### Basic symptoms

In addition to PLE, a second group of unusual experiences predictive of SMI have been identified; the so called basic symptoms (BS), which describe subjectively perceived deficits and abnormalities in multiple domains (perception, cognition, language, feelings) and often represent early manifestations of SMI. BS have been shown to strongly and specifically predict the development of schizophrenia 5-to-10 years later [13],[67],[68]. Since BS precede SMI by at least several years and are distressing and impairing in their own right, they represent a potential target for PEI. Indeed, a psychosocial intervention targeting BS reduced the risk of developing SMI in a clinical high-risk sample [69]. BS have not yet been evaluated in familial high-risk setting and it is unknown if they are more common in offspring of parents with schizophrenia or mood disorders.

#### Functional somatic symptoms

*Functional somatic symptoms* (FSS) include stomach aches, headaches, eye problems and other physical complaints with no known medical cause. FSS in childhood have been found to predict adult depression more strongly than childhood depressive symptoms. [70] FSS are associated with familial risk for depression and bipolar disorder and prospectively predict onset of major mood disorders [71]. FSS also predict onset of psychotic disorder among prodromal subjects [72]. These lines of evidence converge to suggest that FSS may be relatively early and nonspecific antecedents to multiple types of SMI. FSS can be effectively targetted with CBT and mindfulness-based interventions in adults [73],[74] and in children [75].

#### Sleep problems

*Sleep problems* are another common and nonspecific predecessor for a range of mental and physical health problems. Sleep problems in childhood are prospectively associated with a range of mental health problems in adolescence and adulthood, including depression and bipolar disorder [11],[76],[77]. Sleep problems respond well to brief CBT interventions [78]. Therefore, sleep problems may be an early and modifiable antecedent of SMI.

#### Cannabis use

*Cannabis use* is another potential target of early interventions. Regular use of cannabis in adolescence predicts psychotic and mood disorders and deterioration of intellectual functioning [79]-[81]. The use of cannabis and other drugs can be effectively reduced through brief cognitive-behavioural interventions targeted at individual temperamental risk factors for drug use [82].

#### Cognitive delay

Impaired cognitive function and delayed cognitive development are important predictors of SMI [14],[83]-[87]. The predictive significance of cognitive ability may depend on its development: while stably low cognitive ability predicts a wide range of adult mental disorders [14],[83],[84],[86], a progressive decline below one’s projected trajectory may be a relatively specific antecedent to schizophrenia [85],[86]. The relationship between cognitive ability and bipolar disorder is complex: while both poor and excellent cognitive ability in childhood predicts bipolar disorder [14],[88],[89], patients with bipolar disorder and their relatives perform on average worse than controls on cognitive tests [90]-[92]. Low cognitive performance predicts psychosis among subjects at high clinical risk [93]-[99]. This prediction holds across most domains of cognitive ability, but verbal learning and verbal memory show the most robust effects [93],[97],[99].

### Assessments

Separate research teams assess offspring and parents. Both biological parents are assessed. Researchers assessing the offspring are blind to the diagnosis of the parents and vice versa. Parents and offspring are discouraged from discussing details of assessment with each other. All assessors are blind to allocation to interventions and participants are specifically instructed not to mention study intervention to the assessors. Parents and offspring are assessed concurrently to optimize the use of their time.

#### Parent assessment

We assess both biological parents for current and lifetime mental illness with the Structured Interview for DSM-5 diagnoses (SCID-5) [100]. In addition to diagnoses, we collect information on the age of onset, course and severity of each disorder, medical illness, medication, demographic variables and socioeconomic status. We assess family history of mental illness up to third degree relatives with Family History – Research Diagnostic Criteria (FH-RDC) [101]. We measure the current level of psychopathology and well-being with the Everyday Feeling Questionnaire, which enables self- and partner-report [102].

#### Offspring assessment

At baseline and annual follow-ups, we collect demographic information, information on risk factors, antecedents to SMI, full range of psychopathology, cognitive abilities, activities and quality of life. Questions on psychopathology are balanced with positively phrased questions on free time activities and quality of life, to dilute the negative focus of symptom-targeted questions. Initial assessment covers the lifetime psychopathology and risk factor exposures. Follow-up assessments focus on the 12 months period since the last assessment. The same assessments are carried out in FHR and CO. Offspring assessments are listed in Table 2 and described below.

**Table 2:** **Offspring assessments**

Domain	Method	Source	Age
**Descriptive variables**			
Demographics	Questionnaire	Parent	0-25
Socioeconomic status	Questionnaire	Parent	0-25
	FAS	Offspring	7-25
Height, weight, head & waist circumference	Measurement	Offspring	0-18
Pubertal status	Questionnaire	Offspring	9-16
**Psychopathology**			
Diagnosis	K-SADS	Offspring	5-21
	SCID-5	Offspring	18-25
General psychopathology	CBCL	Parent	5-15
	CBCL-YSR	Offspring	11-17
Personality risk factors	SURPS	Offspring	11-15
Mood state	MFQ	Offspring	7-17
Self-control, frustration tolerance	TOF	Rater	3-25
Substance use	DUSI-R	Offspring	9-25
**Functional outcomes**			
General functioning	CIS	Rater	9 - 17
General, role and social functioning	GAF, GF-R, GF-S	Rater	11-25
Quality of life	CHQ	Parent	5 - 18
	CHQ	Offspring	9 - 18
	QOL	Offspring	18 - 25
Activities and milestones	Questionnaire	Parent	0 - 17
		Offspring	9 - 25
**Affective lability**			
Affective lability	CALS-P	Parent	5-16
Affective lability	CALS-C	Offspring	13-16
Affective lability	ALS	Offspring	17-25
Self-control, frustration tolerance	TOF	Rater	3-25
**Anxiety**			5-25
Anxiety (disorders)	K-SADS/SADS-P	Offspring	5-16
Anxiety (dimension)	CBCL	Parent	11-18
Anxiety (dimension)	CBCL-YSR	Offspring	3-5
Anxiety (dimension)	S-CAS	Parent	5-17
Anxiety (dimension)	S-CAS	Offspring	8-17
Anxiety (dimension)	SCARED	Parent	8-25
Anxiety (dimension)	SCARED	Offspring	8-25
**Psychotic symptoms**			
Psychotic-like experiences	Funny Feelings	Offspring	7-18
Psychotic symptoms	K-SADS	Parent	7-18
Attenuated psychotic symptoms	SIPS	Offspring	12-25
Psychotic symptoms	PANSS	Offspring	14-25
**Basic symptoms**			
Basic Symptoms	SPI	Offspring	6-25
**Sleep**			
Sleep	CSHQ	Parent	4-12
	SSR	Offspring	6-14
	SSHS	Offspring	15-17
	PSQI	Offspring	18-25
**Functional somatic symptoms**			
Somatic symptoms	CBCL	Parent	5-15
Somatic symptoms	CBCL-YSR	Offspring	11-17
**Cognitive ability & development**			
General cognitive ability	WPPSI	Offspring	3-5
	WASI	Offspring	6-25
Attention/Processing	Digit-Symbol Coding	Offspring	6-25
Verbal learning and memory	CVLT	Offspring	6-15
	Story recall (CMS)	Offspring	16-25
	Logical Memory (WMS)	Offspring	8-25
Non-verbal memory	BVRT	Offspring	6-25
Executive function, working memory	Letter-Number Sequencing	Offspring	6-25
Spatial working memory	CANTAB	Offspring	5-25
Verbal fluency	D-KEFS	Offspring	7-15
	COWAT	Offspring	16-25
Planning, visuospatial organization	ROCF	Offspring	11-25
Emotional decision making	CANTAB	Offspring	8-25

Abbreviations: Abbreviations: FAS = Family Affluence Scale; K-SADS = Kiddie Schedule for Affective Disorders and Schizophrenia SCID-5 Structured Clinical Interview for DSM-5 Disorders; CBCL = Child Behaviour Checklist; CBCL-YSR = Child Behaviour Checklist - Youth Self Report; SURPS = Substance Use Risk Profile Scale; MFQ = Mood and Feelings Questionnaire; TOF = Test Observation Form; DUSI-R – Drug Use Screening Inventory – Revised; CIS = Columbia Impairment Scale; GF-R = Global Functioning: Role; GF-S = Global Functioning: Social; GAF = Global Assessment of Functioning; CHQ = Child Health Questionnaire; QOL = Quality of Life Short Form; CALS = Child Affective Lability Scales; ALS = Affective Lability Scales; SCAS = Spence Child Anxiety Scale; SCARED = Screen for Child Anxiety Related Disorders; SIPS = Structured Interview for Prodromal Symptoms; PANSS = Positive and negative Symptom Scale; SPI = Schizophrenia Proneness Instrument; CSHQ = Children’s Sleep Habits Questionnaire; SSR = Sleep Self Report; SSHS = School Sleep Habits Survey, PSQI = Pittsburgh Sleep Quality Index; WPPSI = Wechsler Preschool and Primary Scale of Intelligence, WASI = Wechsler Abbreviated Scale of Intelligence; CVLT = California Verbal Learning Task; WMS = Wechsler Memory Scale; BVRT = Benton Visual Retention Task; D-KEFS = Delis Kaplan Executive Functioning System Verbal Fluency Index; COWAT = Controlled Oral Ward Association Test; CANTAB = Cambridge Neuropsychological Test Automated Battery.

#### Descriptive variables

We measure socioeconomic status as parent education, occupation, wealth, income, area of residence, living arrangements (rented/owner occupied; ratio of bedrooms to persons) [103], and with the Family Affluence Scale [104],[105]. We also record the number of children in the household, their sex and ages.

#### Psychopathology

We establish current and lifetime DSM-5 diagnoses in the offspring with a semi-structured diagnostic interview, the Kiddie Schedule for Affective Disorders and Schizophrenia - Present and Lifetime version (K-SADS-PL), adapted for DSM-5 [106],[107], with best estimate diagnoses established in consensus meetings involving psychiatrists blind to the diagnoses of parents. Information provided by parents and by the offspring is submitted for these meetings after checking that it is free of any indication of parental diagnosis or intervention allocation. In offspring aged 18 or over, we use both K-SADS (to cover childhood diagnoses retrospectively) and SCID-5 at baseline and SCID-5 on follow-ups. For each disorder, we establish the age at onset, course and severity. In addition to diagnostic interviews, we obtain self- and parent-report continuous measures of psychopathology [108],[109].

#### Functioning and quality of life

We measure quality of life with the Child Health Questionnaire (CHQ), parent- and young person report, in 5 - 18 year olds [110]-[112] and with the Quality of Life Enjoyment and Satisfaction Questionnaire-Short Form (QOL) in adults (>18 years). We measure functional outcomes using the Global functioning: Role and Social scales [113], Columbia Impairment Scale (CIS), parent and youth report, and a list of developmental milestones (school exams, driver’s licence, first summer job, …).

#### Affective lability

We assess AL with the child - and adult versions of the Affective Lability Scales (ALS) [32],[33],[114]. The Child ALS is rated by parents of 7 to 16 year olds [32]. The self-report child version is used from age 12 and the adult version from age 17 onwards [33],[114]. Where more than one measure is available from the same assessment (e.g. parent and self-report), we consider the higher score unless there is a reason to doubt the validity of the higher scoring informant [115],[116]. We define the antecedent ‘affective lability’ as a score of 1 standard deviation or more above the mean of a normative sample [38].

#### Anxiety

We assess anxiety disorders and symptoms with semi-structured diagnostic interviews and dimensional measures. We define the antecedent ‘anxiety’ as a diagnosis of an anxiety disorder (generalized anxiety disorder, social phobia, agoraphobia, panic disorder, separation anxiety disorder, specific phobia, obsessive compulsive disorder or posttraumatic stress disorder) with K-SADS or SCID-5, or a score above the high-specificity cut-off (≥30) on the Screen for Child Anxiety Related Emotional Disorders (SCARED) [109]. In children below age 9, anxiety is defined as a score 1 standard deviation or more above a normative sample on the parent-reported Spence Children Anxiety Scale (S-CAS) [117]. Following the established standard in child psychiatry, we consider anxiety present if reported by either the parent or the child unless there is a reason to doubt the veracity of a positive report [115],[116],[118],[119].

#### Psychotic-like experiences

We measure PLE with several validated instruments. In offspring aged 7 to 21 years, we use the ‘Funny feeling’ (FF) interview where the psychotic character of initial self-report is corroborated with probes about the nature and context of the experience [66],[120]. We record frequency, distress, impairment and appraisal (internal/external, significant/not-significant) for each symptom. An independent clinical evaluator curates the verbatim transcription of each unusual experience and rates its psychotic character as none, probable or definite. Only PLE curated as ‘definite’ qualify as an antecedent. In addition, we assess psychotic symptoms with parent- and youth-report in the K-SADS interview, consensus-rated by an independent certified child and adolescent psychiatrist. In participants aged 12 and more, we also assess psychotic symptoms with the Structured Interview for Prodromal Symptoms (SIPS) [121]. The antecedent PLE is present if one or more symptoms are independently curated or clinician consensus-confirmed as definitely psychotic.

#### Basic symptoms

We assess basic symptoms with the Schizophrenia Proneness Instrument Child and Youth version. (SPI-CY) [122],[123]. We define the antecedent ‘basic symptoms’ as fulfilling criteria for one or both of the high-risk basic symptom profiles that were shown to predict schizophrenia with high specificity: Cognitive Perceptive basic symptoms (COPER) requiring a severity rating of 3 or more on SPI-CY for one or more of the 10 most strongly predictive cognitive or perceptual domain symptoms, or Cognitive Disturbance (COGDIS) requiring 2 of 9 cognitive/perceptual symptoms scored 3 or higher, as recommended by the measure authors [122],[123].

#### Functional somatic symptoms

We measure FSS with the somatic subscale of the parent-report Child Behavioural Checklist (CBCL) and the self-report of the Youth Self Report (YSR) [108].

#### Sleep problems

We assess sleep problems with the parent-report Children’s Sleep Habits Questionnaire (CSHQ) [124], and several self-report measures for different age groups, including the Sleep Self Report (SSR) [125], School Sleep Habits Survey (SSHS) [124] and the Pittsburgh Sleep Quality Index (PSQI) [126].

#### Cannabis use

We assess the frequency of cannabis and other drug use with the self-report Drug Use Screening Inventory - Revised (DUSI-R) [127] in addition to diagnostic interviews.

#### Cognitive delay

We assess both general cognitive ability and specific aspects of cognitions that are relevant to SMI. We list the cognitive tests and applicable age in Table 2. We assess general cognitive ability with the Wechsler Abbreviated Scale of Intelligence, Second Edition (WASI-II), which which contains four tests (vocabulary, block design, similarities and matrix reasoning) and is normed to provide a standardized full scale general cognitive ability score for subjects from 6 years onwards [128]. In participants aged 3 to 5, we use the Wechsler Preschool and Primary Scale of Intelligence (WPPSI-III), which is normed for ages 2.6-7.3 [129]. In addition, we include specific tests selected based on their ability to discriminate individuals at high familial or clinical risk for SMI from controls and predict onset of SMI. These include attention and speed (digit-symbol coding test DSCT subtest from the WPPSI, WISC-IV, WAIS-IV), working memory (Letter Number Sequencing; Digit Span backward subtests from the WISC-IV, WAIS-IV), spatial working memory (CANTAB), verbal learning and memory (California Verbal Learning Test - Children’s Version, CVLT-C), logical memory (story recall from Wechsler Memory Scale – Revised or Children’s Memory Scale, LM), verbal fluency (Delis Kaplan Executive Functioning System Verbal Fluency Index, D-KEFS; Controlled Oral Word Association Test, COWAT), emotional decision making (Cambridge Gambling Task (CGT), planning/visuospatial organization in executive function (Rey-Osterrieth Complex Figure, ROCF), and visuospatial memory and organization (Benton Visual Retention Test [BVRT]) [85],[90],[93]-[95],[97]-[99],[130]-[132]. Tests are administered by master-level psychologists, trained and supervised by doctoral-level clinical neuropsychologists. Where alternative forms are available (CVLT, COWAT, D-KEFS, BVRT), they are alternated in a fixed order that is the same in FHR and CO. We construct an overall standardized score as a mean of standard scores from the administered tests, providing a general measure of cognitive ability weighted towards the cognitive domains that are most predictive of SMI. We define cognitive impairment as performance 1 standard deviation (corresponding to 15 points on a standardized scale) below age-appropriate population norms. We define cognitive delay as a decline of 2/3 standard deviation (corresponding to 10 points on a standardized scale) or more against own trajectory estimated from previous measurements.

### Follow-up and retention of participants

The validity of longitudinal study results depends on retention rates. We build on experience from cohort studies that achieved long-term retention rates over 90% [11],[79],[120],[133]-[139]. We employ strategies to minimize attrition including regular friendly and non-stigmatising contact (updates, newsletters and greeting cards), requesting multiple contact routes and repeated attempts to contact hard-to-reach individuals [138]. We provide a welcoming environment with seamless completion of assessments without unnecessary hassle. We reimburse participants for their time and we support their transport costs. Our target is 90% retention over 3 years.

### Interventions

We preferentially consider low-burden, low-risk interventions that are proportionate to the relatively mild antecedent psychopathology and are likely to be acceptable to a large proportion of non-treatment seeking participants and their families. The primary focus is on psychological and nutrition supplement interventions.

For children below the age of 9, the interventions will primarily target parents and carers, with an optional involvement of the child participant. Parent skill training has strong evidence for efficacy in conditions characterized by affective lability and anxiety [54]. Parent skills training can be combined with cognitive training for children to address cognitive delay and attentional problems. [140]

For youth aged 9 and above, the psychological interventions will focus on the young individual, with optional involvement of parents or carers. The first such intervention will involve the youth learning skills for emotional wellbeing, including emotional self-understanding, problem solving, present moment focus, distress tolerance, reality testing, activity scheduling, and healthy sleeping, following the principles of CBT. The intervention is modular and adapted to the individual through a combination of core and optional modules, potentially addressing multiple antecedents [141]. There is evidence that CBT in childhood and adolescence has long-term positive impact on mental health [142].

Further psychological intervention may address temperamental risk factors that put an individual at risk for drug use and other risk taking behaviours. There is evidence that such interventions have lasting effects on multiple domains of mental health and well-being [82].

Another type of safe and potentially effective interventions are dietary supplements, such as polyunsaturated fatty acids, vitamin D and choline, which have evidence for beneficial effects on neurodevelopment [25],[143],[144]. The selection and development of interventions are ongoing and will take into account the evolving evidence base for safety and efficacy.

### Outcome measures

The primary short-term outcome for intervention studies within FORBOW is the persistence of antecedents in the assessments following the offer of intervention. The primary long-term outcome is the development of SMI. Secondary outcomes are dimensional measures of functioning, distress, psychopathology and quality of life and diagnosis of any mental disorder on follow-ups.

### Data analysis strategy

The analysis of outcomes will follow the intention-to-treat principle [145]. Effects of interventions on antecedent persistence will be tested with lagged effect binomial regression models. Long-term effects of interventions on the risk of SMI onset will be tested in proportional hazard survival models. Clustering of siblings within a family will be accounted for by hierarchical random effects of individual and (where more than one sibling from same family are included) of family or estimation of standard errors robust to clustering within families. Missing data on covariates will be handled with multiple imputation [146], so that missing covariates do not reduce the number of subjects available for analyses. Missing data on primary outcomes (antecedents) will not be imputed [147].

## Discussion

### Ethical aspects

FORBOW assessments involve safe established procedures and participation in FORBOW does not limit participants in accessing any type of care. However, FORBOW includes psychiatric assessments and offers of interventions to young individuals, who are not presently seeking treatment. Therefore, it is essential to ensure confidentiality and minimize the risk of stigmatization (including self-stigmatization). We collaborate with organizations of people with lived experience of mental illness and communication specialists in the area of mental health to optimize acceptability and minimize risks. The inclusion of control families from the general population and sensitive communication ensure that participation in the study is not associated with a ‘risk’ label. We ask all parents and offspring who have the capacity to provide written consent after the study procedures are explained and written information is provided. We ask parents or guardians for written authorization for participation of offspring who may not have the capacity to provide consent. This includes consent to access electronic health-care related data through linkage with health card numbers, and consent to be contacted for additional research studies, including studies of interventions. We ask offspring who lack the capacity to provide consent for a verbal assent and we only include them if both consent and assent are provided.

We acknowledge that research diagnosis does not equal the need for treatment and we do not actively provide feedback on diagnoses and test results. We handle requests from participants or families for individual feedback on case-to-case basis with involvement of a licensed psychiatrist. Any feedback respects confidentiality of individual participants: information provided by offspring is not disclosed to parents unless such disclosure is necessary to prevent significant harm. Diagnosis of parents will not be disclosed to the offspring. The study protocol has been approved by the Capital District Health Authority Research Ethics Board, the IWK Health Centre Research Ethics Board, health authorities across Nova Scotia, and the Department of Community Services, Nova Scotia, Canada.

### Conclusion and directions

Through a combination of familial history and antecedents, FORBOW provides an opportunity to bring early intervention efforts into a younger age group compared to interventions in prodromal stages of SMI. Indirect evidence suggests that earlier interventions may have greater beneficial influence. However, only the long-term results of FORBOW and similar studies will provide the definite answer on whether earlier is better. Thanks to random selection of eligible individuals for interventions, FORBOW will experimentally test the role of early antecedents in the etiology of mental illness. Even with accelerated cohort design, the main results will take a decade to emerge.

Webpage: http://www.forbow.org.

## Authors’ original submitted files for images

Below are the links to the authors’ original submitted files for images. Authors’ original file for figure 1 Authors’ original file for figure 2
