# Supplementary figures and images for: A familial risk enriched cohort as a platform for testing early interventions to prevent severe mental illness
Source: BMC Psychiatry. 2014 Dec 2;14:344. doi: 10.1186/s12888-014-0344-2 (PMC4267051; doi:10.1186/s12888-014-0344-2)

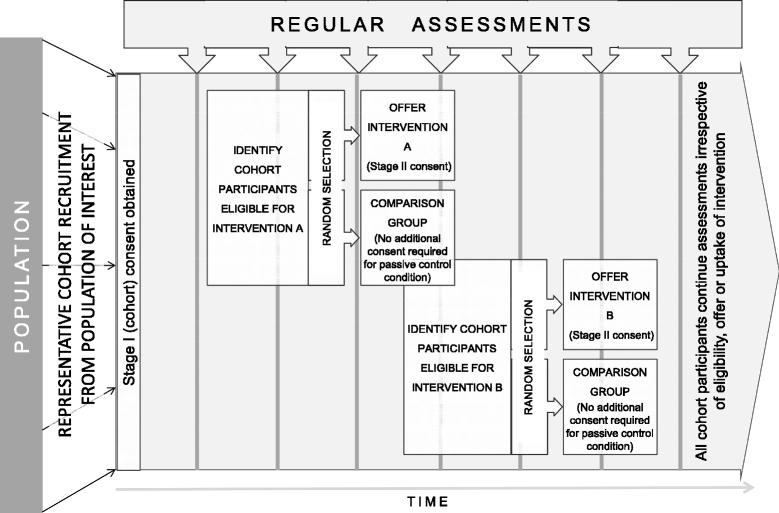

Supplement: Supplementary file 1 — Authors’ original file for figure 1 [file 12888_2014_344_MOESM1_ESM.gif]

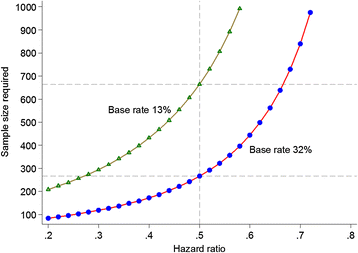

Supplement: Supplementary file 2 — Authors’ original file for figure 2 [file 12888_2014_344_MOESM2_ESM.gif]
